# Microbiota assembly in *Zostera marina* during early host development across controlled growth experiments

**DOI:** 10.1128/msystems.00722-26

**Published:** 2026-07-27

**Authors:** Gina Chaput, Emma A. Deen, Eric Q. Pham, Sarah Crystal, Andrew Matayoshi, Peter Andeer, Trent R. Northen, Jonathan A. Eisen, John J. Stachowicz, E. Maggie Sogin

**Affiliations:** 1 Genome Center, University of California Davis 70006, Davis, California, USA; 2 Department of Evolution and Ecology, University of California Davis 166622, Davis, California, USA; 3 Environmental Genomics and Systems Biology, Lawrence Berkeley National Laboratory 1666, Berkeley, California, USA; 4 The DOE Joint Genome Institute, Lawrence Berkeley National Laboratory 1666, Berkeley, California, USA; 5 Department of Medical Microbiology and Immunology, University of California Davis 272916, Davis, California, USA; 6 Department of Molecular Cell Biology, University of California Merced 33244, Merced, California, USA; Boise State University, Boise, Idaho, USA

**Keywords:** plant–microbe interactions, microbiome, microbial ecology

## Abstract

Seagrass restoration practices are evolving to leverage microbiome applications, similar to agricultural systems that have demonstrated how targeted microbial communities enhance crop resilience in challenging environments. While adult seagrass microbiome research has expanded significantly, research on the seed microbiome remains critically understudied. This gap is important given that seeds represent a large portion of restoration efforts. Advancing seed microbiome research requires standardized experimental systems for controlled plant–microbe interaction studies, which are currently lacking in seagrass research. Here, we tested fabricated ecosystem devices (EcoFAB 2.0) as a standardized system for growing *Zostera marina* (eelgrass) seedlings, enabling a controlled study of aquatic plant–microbe interactions. Using these chambers, we addressed three key questions: (i) can we reliably grow eelgrass in a controlled laboratory setting, (ii) can we manipulate eelgrass microbiota assembly and its long-term trajectory, and (iii) can we detect shifts in the microbiota during plant development (host filtering)? Host morphology measurements and 16S rRNA gene amplicon sequencing were used to track microbiota assembly across three early developmental stages of the host. Because plants were grown in a sterile environment, surface sterilization of seeds (bleach and ethanol) removed epiphytes without disturbing the shared endophytic community, yet microbiota composition remained divergent at Stage 6 (143 differentially abundant ASVs), indicating that seed coat epiphytes make a lasting and distinct contribution to assembly trajectory. We also identified 26 stage-specific indicator ASVs across eelgrass development, suggesting stage-specific microbial associations during seedling establishment. This work demonstrates the potential for targeted manipulation of the microbiome in seagrass for restoration efforts.

**IMPORTANCE:**

Using the Fabricated Ecosystem 2.0 (EcoFAB 2.0), we were able to successfully control the microbial environment of eelgrass, *Zostera marina*, resulting in the reduction of epiphytes and maintaining low microbial diversity across plants without compromising the morphology and growth of seedlings. Our findings advance the marine plant model system, *Z. marina*, by identifying taxonomic indicators across life stages. This work lays the foundation for a targeted understanding and application of microbiomes for seagrass restoration, bridging the critical knowledge gap between agricultural seed microbiome success and marine restoration applications.

## INTRODUCTION

Seagrasses, including *Zostera marina* (also known as eelgrass), are foundation species that occur along coastlines and serve as nurseries for commercial and ecologically important organisms, protect coastlines from erosion, and drive biogeochemical cycling of nutrients (1, 2). Human activities are driving declines in seagrass meadows, which jeopardize the services they provide to coastal communities (3). Efforts to restore deteriorated meadows or establish new habitats have had mixed success, with research reporting varying outcomes across different approaches and locations (4, 5). To achieve meaningful progress, it is critical to develop solutions that protect current seagrass populations, work alongside local communities to apply methods that work for both seagrass and local economies, and develop solutions that “future-proof” seagrass populations (6). The resilience of seagrass beds could be enhanced through a range of approaches, including selective breeding, acclimatization, and food web manipulations. Additionally, the possibility of leveraging the microbiome to promote plant resilience and resistance to stressors has been proposed (6, 7). Terrestrial agroecosystems have a long history of application of microbiome science to increase crop yields, and these approaches have recently been piloted in some marine systems as well (e.g., corals and mangroves) (8, 9), suggesting that microbes have untapped potential in seagrass restoration and conservation.

Seed microbiomes of crops, such as wheat, maize, and tomatoes, provide protection, promote germination, and aid in nutrient uptake as the seed begins to develop (10–12). The growing recognition of seed microbiomes’ importance in plant success has led to the development of synthetic microbial communities that can be inoculated onto seeds for use in agriculture (13, 14). However, much less is known about the role of microbial species in promoting seagrass growth or seed development. Current understanding of the *Z. marina* microbiomes is largely based on adult plants (15–23). It is unclear how the microbiota assembles on seagrass seeds and seedlings and the functional roles of individual members once established (16, 24). To assess the potential of microbiome treatments to facilitate restoration and conservation, we must first understand the assembly and roles of different microbial taxa in early post-germination processes. This includes determining their impacts on germination success and seedling survival.

Experimental approaches to understanding seagrass seed microbiota assembly pose several logistical challenges given the aquatic nature of their environment and their growth across a sediment–water interface. Such work would be greatly facilitated by developing sterile, fabricated system approaches that enable precise manipulation of the plant microbiome during seagrass development. Multiple mesocosm chamber designs have been developed for studying terrestrial plant–microbe interactions in the rhizosphere (see Yee et al. for a comprehensive review [25–27]). Similarly, phyllosphere research has adopted various platforms alongside traditional approaches, including petri dishes, tissue culture boxes, and FlowPots/GnotoPots (28). In contrast, seagrass research has relied on a limited suite of culture systems: petri dishes for seed germination (29), glass bottles for seedling cultivation (30), and flow-through tanks or two-compartment hydroponic chambers for adult plant growth (15, 31). Here, we adapt the Fabricated Ecosystem 2.0 (EcoFAB), a self-contained and dual-compartment platform (26, 32), to grow *Z. marina* in a fully submerged environment while tracking microbiota assembly throughout plant development. We successfully altered the microbiota of *Z. marina* through the removal of the seed epiphytic microbiota, maintaining consistently low microbial diversity across plants without compromising seedling growth dynamics. These chambers allowed us to address the following questions: (i) can we reliably grow eelgrass in a controlled laboratory setting, (ii) van we manipulate the eelgrass microbiota assembly and its long-term trajectory successfully, and (iii) can we detect shifts in the microbiota during plant development (i.e., host filtering)?

## MATERIALS AND METHODS

### Seed collection and treatment

*Z. marina* flowering shoots were collected from Bodega Bay, CA (38°20′00.6″N 123°03′34.7″W), in June, July, and August of 2023. At Bodega Bay, CA, in 2022, the yearly salinity was ~32–33 PSU (33). This is consistent with historical averages recorded of 32–34 PSU for this area (34). To allow developing seeds to mature while preventing sediment-associated microbiota contamination, flowering shoots with intact rhizomes were rinsed and placed in 500 μm nylon mesh bags, then held in flow-through tanks at the UC Davis Coastal and Marine Sciences Institute’s Bodega Marine Laboratory until September (average salinity ~32–33 PSU). Seeds were sorted, and any with visible damage, pathogen growth (e.g., *Phytophthora*; defined as white filaments protruding from the seed) (35), or softness under pressure were removed (36). The resulting 1,017 seeds were divided into reduced (*n* = 500) and intact microbiota (*n* = 517) treatment groups. To reduce the epiphytic microbiota, we adapted surface sterilization techniques used for terrestrial seed microbiome studies (13, 37). Although we acknowledge that our treatment does not represent full sterilization, our approach is analogous to those used in terrestrial plant studies and termed “seed surface sterilization,” as it removes native bacterial epiphytes from the seed coat (38–42). This method is commonly used in terrestrial seed studies to isolate the effect of plant endophytes (within-tissue microbes), and we interpret our results similarly: reducing the microbiota on seeds prior to germination allows us to examine how the endophytic microbiota colonize the entire plant during development. We refer to seeds that were surface sterilized (the procedure) as having a “reduced microbiota” (the result) throughout this study. Seeds were surface sterilized using a single wash each of 95% ethanol (15 s), 5% bleach (3 min), and 70% ethanol (2 min), followed by three washes with filter sterilized (0.22 µm) artificial seawater (Instant Ocean; 32 PPT; 30 s each). Seeds with intact microbiota were shaken with filter-sterilized synthetic seawater (three washes, 30 s each) to mimic some of the scarification that the surface sterilization procedure may have caused (43, 44). To confirm bacterial load differences between the treatment groups, we tested our methods before the experiment by plating the third wash of artificial seawater from each treatment group onto marine broth agar and found that the reduced microbiota group had no colony-forming units, whereas the control group did.

**Fig 1:**
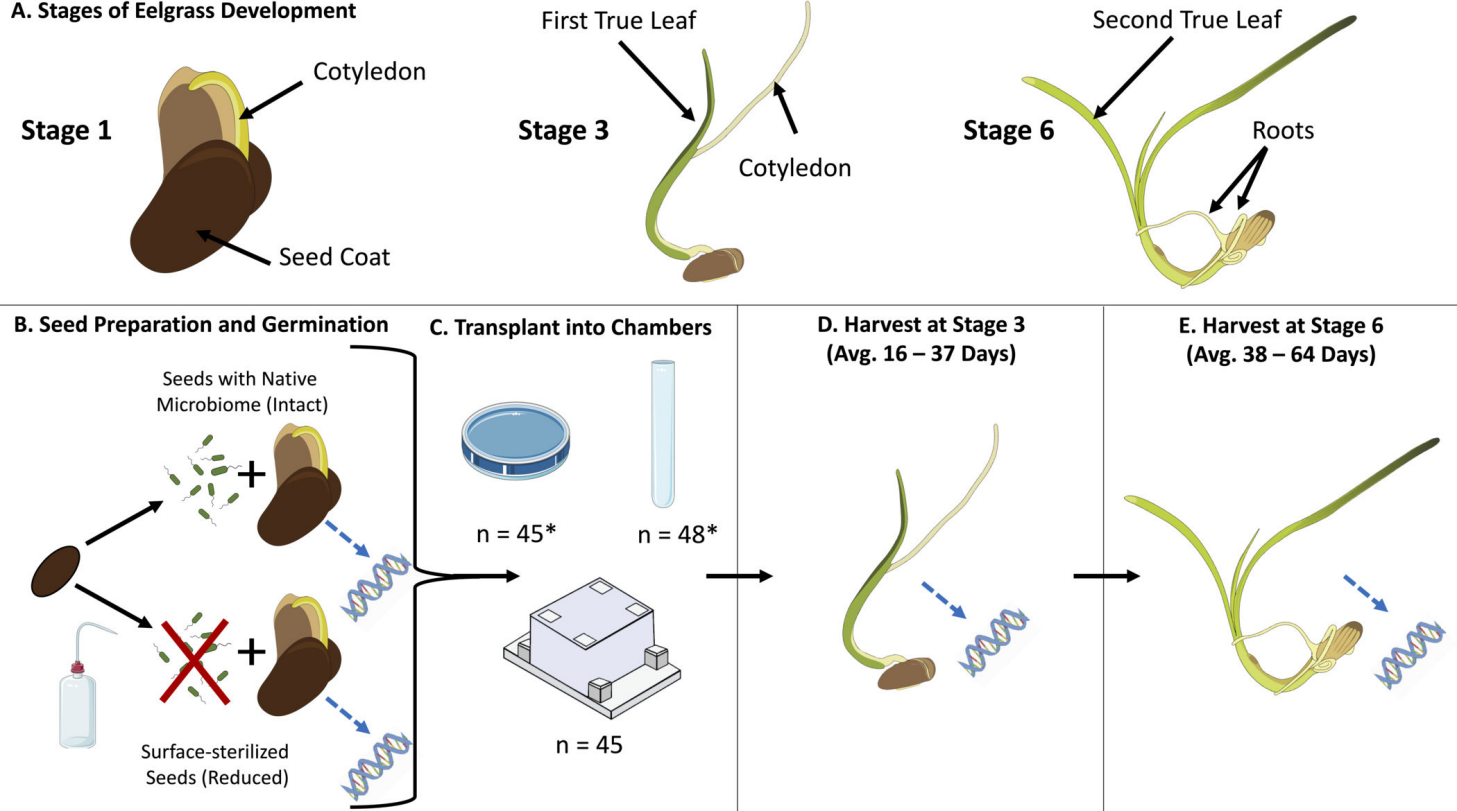
Experimental design and sample breakdown. (**A**) Stages of eelgrass development were based on descriptions by Xu et al. (29). Stages of interest were stages 1 (initial germination, before introduction to chambers), 3 (when the first true leaf had developed), and 6 (when the cotyledon had senesced and abscised; roots had formed; and the second true leaf had appeared). (**B**) Seeds were prepared to have either reduced or intact microbiota prior to germination. (**C**) Following germination, plants were randomly selected to be destructively harvested at Stage 1 (indicated by blue dashed arrows) or introduced to EcoFAB 2.0 devices, petri dishes, or test tubes. (**D**) Plants were harvested at either Stage 3 or (**E**) 6. Each plant was photographed, and morphometric measurements were taken using ImageJ. Following imaging, DNA was extracted from whole plant samples for 16S rRNA gene amplicon sequencing. * indicates that chambers were shaken for 30 min per day.

The reduced and intact seeds were allowed to either germinate in the dark or were under a 12 h:12 h light:dark cycle. All seeds prior to germinating were kept at 15°C and placed into individual petri dishes filled with 8 mL of filter-sterilized (0.22 µm) synthetic seawater (Instant Ocean; 32 PPT) and 5 mL of hydrogel beads (14G needle) (45). By having each seed in its own petri dish, we avoided the cross-contamination of potential disease, microbiota, and seed exudates. Seeds were checked every 3–4 days for initial germination, pathogen or algal overgrowth, and mortality. Of the 1,017 seeds that were individually monitored, 186 seeds germinated (18%), which is within the range of observed germination success at similar salinities under laboratory conditions (29). After sampling for microbiota composition at initial germination (Stage 1; Fig. 1A; *n* = 12), a subset of the seeds were placed in either the EcoFAB (*n* = 45), petri dish (*n* = 45), or test tube (*n* = 48) chambers and allowed to grow until the plant developed the first true leaf (Stage 3; Fig. 1A) or when the cotyledon senesces and abscises; roots are formed; and at least two true leaves have appeared (Stage 6; Fig. 1A; summary of seed sample size in Table S1). Germinants that died between stages (Table S1) were excluded from the analysis, as they provided no information about stage-specific microbiota. All chambers were kept at 15°C and maintained on a 12 h:12 h light:dark cycle. To avoid an oxygen gradient forming in the smaller-volume petri dishes and test tubes (8 and 10 mL, respectively), chambers were gently shaken for 30 min per day at 180 RPM.

### EcoFAB 2.0 preparation for eelgrass cultivation

EcoFAB 2.0 devices were assembled as previously described with minor modifications (46). Both the upper compartment and base of the EcoFAB 2.0 were filled with filter-sterilized (0.22 µm) synthetic seawater (Instant Ocean; 32 PPT). Approximately 5 mL of hydrogel beads soaked in synthetic seawater was added to the base to mimic sediments (45). To maintain water-tight seals, a 1/32″-thick square silicone gasket was cut and placed between the EcoFAB upper compartment and base. Additional rubber washers were placed with the screws at each corner of the chamber to accommodate the gasket thickness. Test tube and petri dish chambers were prepared similarly, with ~5 mL of hydrogel beads in the bottom and topped with synthetic seawater.

### Eelgrass seed and seedling sampling and measurements

Across treatments, seeds and seedlings were randomly selected to be destructively harvested before introduction to the chambers (Stage 1: initial germination), at Stage 3 (first true leaf develops), or Stage 6 (cotyledon senesces and abscises; roots are formed; and at least two true leaves have appeared; Fig. 1; Table S2) of development. For each seedling, each plant was photographed using a dissecting microscope, and the cotyledon (cotyledonary blade, cotyledonary sheath, and axial hypocotyls), leaf, and root lengths were measured using ImageJ (47). Following imaging, samples (entire plants) were stored at −80°C until processed for DNA extractions. Total DNA from the entire plant was extracted using the DNeasy Plant Mini Kit (Qiagen, Hilden, Germany) following the manufacturer’s guidelines.

### 16S rRNA gene library construction and sequencing

DNA was prepared for V3/V4 amplification of 16S rRNA genes using the Zymo Quick-16S Plus NGS Library Prep Kit and unique dual indexes (Zymo Research, CA, USA). Primers used by the Zymo Quick-16S Plus NGS Library Prep Kit are 341F (CCTACGGGDGGCWGCAG, CCTAYGGGGYGCWGCAG; 17 bp) and 806R (GACTACNVGGGTMTCTAATCC; 21 bp). Peptide nucleic acid (PNA) blockers were added to reduce chloroplast and mitochondrial sequence products as previously described (22). PCR conditions were: 95°C for 10 min, 35 cycles (of 95°C for 30 s, 55°C for 30 s, 72°C for 3 min), and 72°C for 6 min. Sequencing was performed on the Illumina NextSeq2000 platform using a 600-cycle flow cell kit to produce 2 × 300 bp paired reads (SeqCoast Genomics, NH, USA). A 30–40% PhiX control (unindexed) was spiked into the library pool to support optimal base calling of low-diversity libraries on patterned flow cells. Read demultiplexing, read trimming, and run analytics were performed using DRAGEN v3.10.12 (48), an onboard analysis software on the NextSeq2000.

### Data analyses for seedling morphology measurements and sequence data

A binomial generalized linear model (logistic regression) was used to determine how seed (i.e., reduced vs. intact microbiota) and light (i.e., dark or a 12 h:12 h light:dark cycle) treatments affected seed germination success. To compare variation in plant growth (cotyledon, leaf, and root length) as a function of seed treatment and chamber type (i.e., test tube, petri dish, and EcoFAB), a combination of parametric (i.e., two-way analysis of variance [ANOVA], mixed-linear effects model) and non-parametric approaches (i.e., Scheirer–Ray–Hare test) was used. For Stage 3 leaf lengths, a linear mixed-effects model (LMM fit by REML) that is robust to heteroscedasticity was applied, while for Stage 3 cotyledon lengths, a Scheirer–Ray–Hare test, followed by a Dunn’s *post hoc* test, was used. Stage 6 leaf measurements were tested with a two-way ANOVA, and root length was tested with a Scheirer–Ray–Hare test, followed by a Dunn’s *post hoc* test. Effects of seed treatment and chamber type on days until the plant developed to either Stage 3 or 6 were tested with a Scheirer–Ray–Hare test, followed by a Dunn’s *post hoc* test. All statistics described above were analyzed in R Statistical Software (v.4.2.1) (49) and RStudio (50) (v.2022.7.1.554) with the vegan (v.2.6-4) (51), rcompanion (v.2.3.0) (52), and dunn.test (v.1.3.6) (53) packages.

16S rRNA gene amplicon sequences were analyzed in R Statistical Software (v4.2.1) (49), with DADA2 (v1.26.0) following the 1.8 Tutorial and Big Data Tutorial (54). Default parameters for DADA2 were used, except the following: at the 5′ end, reads were trimmed 17 bp and truncated at 290 bp (F), while at the 3′ end, reads were trimmed 21 bp and truncated at 200 bp (R). Bacterial ASVs were aligned to the SILVA database (version 138.1) (55, 56) for taxonomic assignment. If ASVs were unassigned at the domain level, they were removed from further analysis. Using phyloseq (v.1.42.0) (57), all chloroplast, mitochondrial, or plant-assigned ASVs were also removed, leaving 6,060 ASVs. Due to varying library sizes, samples were rarefied to 1,593 reads per sample, and ASVs with fewer than two reads across the entire data set were removed from further analyses, resulting in 811 unique ASVs across all samples. Using phyloseq, the Simpson and Shannon diversity indices of the microbiota for each sample were calculated, and significant differences were tested for using two separate two-way Scheirer–Ray–Hare tests: seed treatment × developmental stage and seed treatment × chamber type. *P*-values were adjusted for multiple comparisons using the false discovery rate (FDR) correction. Beta diversity and dispersion were calculated and tested between treatment groups with permutational multivariate analysis of variance (PERMANOVA) and ANOVA, respectively, using both vegan (v.2.6-4) (51) and pairwiseAdonis (v.0.4.1) (58). Using indicspecies (v.1.8.0) (59), ASVs that can be used as indicators for eelgrass development at stages 1, 3, and 6 were identified. The microbiome package (v.1.20.0) (60) was used to calculate the relative abundances for all ASVs, and indicator ASVs were visualized with pheatmap (v.1.0.12) (61). To compare which ASVs were significantly different in relative abundance between seed treatment groups at Stage 6, DESeq2 (v.1.38.3) (62) was used on non-rarefied data (final non-zero ASVs were 2,358). Size factors were estimated using poscounts normalization [estimateSizeFactors(type = “poscounts”)], which is recommended for sparse amplicon data to account for excess zeros. ASVs were considered significantly differentially abundant if they met a dual threshold of FDR-corrected significance (*P*adj < 0.05) and a minimum absolute log2 fold change of 2. To assess microbiota stability between developmental stages, Spearman’s rank correlation was calculated between stages 1 and 6 mean abundances for each of the DESeq2-flagged ASVs analyzed separately for each seed treatment group. The phylogenetic tree of the significant ASVs from the DESeq2 analysis was constructed using Mafft (v.7) (63) and FastTree (v2.2.0) (64) and visualized with ggtree (v.3.6.2) (65).

## RESULTS AND DISCUSSION

### Eelgrass can be grown reliably in a controlled laboratory setting

**Fig 2:**
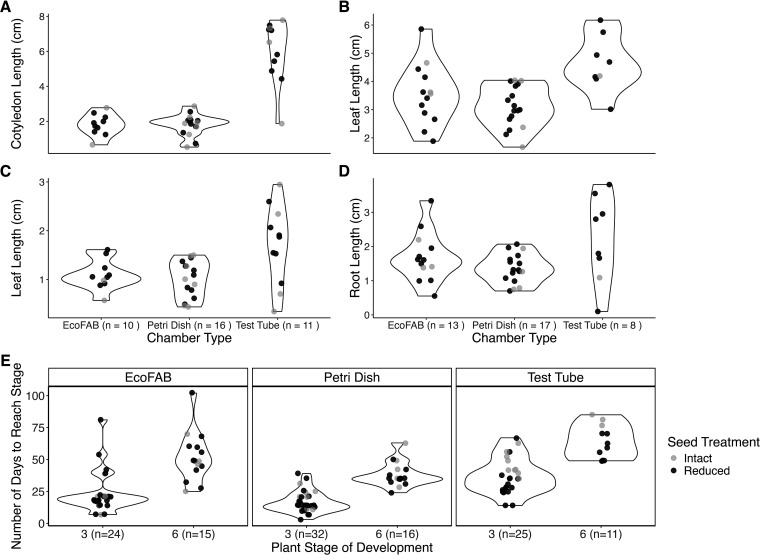
Chamber type, not seed treatment, affects plant morphology and development. Violin plots showing (**A**) cotyledon length (including hypocotyl), (**B**) leaf length at Stage 3, (**C**) leaf length at Stage 6, (**D**) root length at Stage 6, and (**E**) days until development stages 3 and 6 are reached for eelgrass grown in either EcoFABs, petri dishes, or test tubes.

Seeds with a reduced microbiota had higher germination success (23.2%) in comparison to those with an intact microbiota (13.5%; GLM, df = 1,013, *P* = 0.0019). Germination success was not affected by light conditions (GLM, df = 1,013, *P* = 0.4942) or the interaction between light and seed treatment (GLM, df = 1,013, *P* = 0.5973). There was no effect of the seed surface treatment on *Z. marina* morphological development (Fig. 2), including days taken to reach each developmental stages 3 and 6 (Scheirer–Ray–Hare test, *P* > 0.05), cotyledon length at Stage 3 (Scheirer–Ray–Hare test, *P* > 0.05), leaf length at either Stage 3 (LMM, *P* > 0.05) or 6 (ANOVA, df = 36, *P* > 0.05), and root length at Stage 6 (Scheirer–Ray–Hare test, *P* > 0.05). Our findings indicate that while eelgrass with reduced microbiota exhibit increased germination, this does not affect plant development; thus, this is a promising approach for knocking back seed surface microbiota that can increase the chances of establishment of desired innocula.

Plant morphology varied among petri dishes, test tubes, and EcoFABs (Fig. 2). Specifically, Stage 3 cotyledon lengths were significantly longer in test tubes compared to EcoFABs and petri dishes (Scheirer–Ray–Hare test *P* = 0.00010; Dunn’s Test *P* < 0.05). A possible reason for this length difference between chambers is the behavior of the axial hypocotyl, which elongates when in a low oxygen environment, such as when buried within sediment (36, 66). Although both test tubes and petri dishes were gently shaken for 30 min each day, test tubes may have had lower oxygen concentrations than the petri dish or EcoFAB chambers due to their elongated shape and deeper water column. For leaf length, Stage 3 leaves were not statistically different between chambers (LMM, *P* > 0.05), but plants held in test tubes had significantly longer leaves compared to petri dishes and EcoFABs at Stage 6 (ANOVA, df = 35, *P* = 0.00174; Dunn’s test *P* < 0.05). Root length at Stage 6 did not differ between chamber types (Scheirer–Ray–Hare test, *P* > 0.05).

The rate of plant development also differed among chamber types (Scheirer–Ray–Hare test *P* < 0.001 and 0.00028 for stages 3 and 6, respectively). In test tubes, plants reached Stage 3 after an average of 37 days and progressed to Stage 6 in 64 days. In EcoFABs, development to Stage 3 occurred more quickly in 23 days, and Stage 6 was reached in 52 days. Petri dishes showed the fastest development, with Stage 3 reached at 16 days and Stage 6 in 38 days. One potential explanation is that with increased cotyledon length, energy reserves for the seed are diminished (36, 66), which may underlie the delayed development between stages in test tubes. Together, these findings suggest that test tubes are not ideal chambers for examining seedling development due to the likely stress-induced morphologies being exhibited and the large variability in plant development rates among individuals.

**Fig 3:**
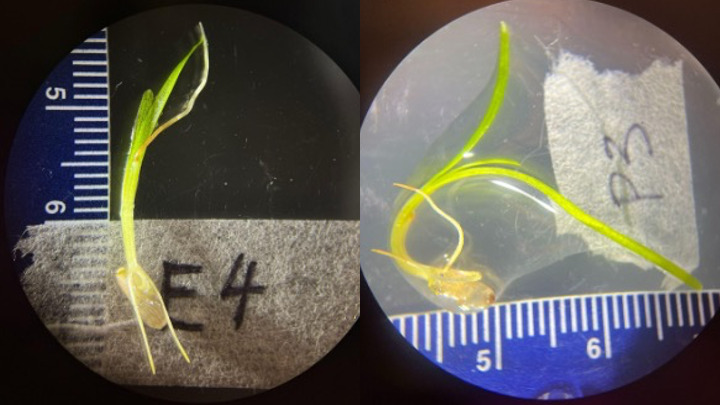
Eelgrass grown in petri dishes (left) displayed greater curvature at the sheath as leaves developed compared to those grown in EcoFABs (right). Representative dissecting microscope images (10× magnification) of eelgrass seedlings (Stage 6) in either petri dishes (left) or EcoFAB 2.0 devices (right). Scale bar = 0.1 mm.

EcoFABs and petri dishes produced seedlings with similar growth rates and length, but we observed that seedlings curved at the sheath as their leaves developed in the petri dishes (Fig. 3). Plant tropisms, such as curving, result from multiple plant behaviors, including hormone production (67), which in turn can affect plant–microbiome interactions (68). These plant responses add uncertainty in interpreting microbial results and limit the representativeness of findings when extrapolated to what is occurring in the field. Future work is needed to test the metabolomic profile of the host between the petri dish and EcoFAB to determine if host metabolites change between devices and whether there are long-term effects on plant development. In addition to changes in metabolic profiles, we also recommend testing how metabolites in petri dishes, which would include exudates from both leaves and roots in the same compartment, would affect microbiota assembly versus the EcoFAB that has two separate compartments that separate the phyllosphere and rhizosphere.

### Eelgrass microbiota assembly can be manipulated with seed surface sterilization techniques and has long-term trajectory effects

**Fig 4:**
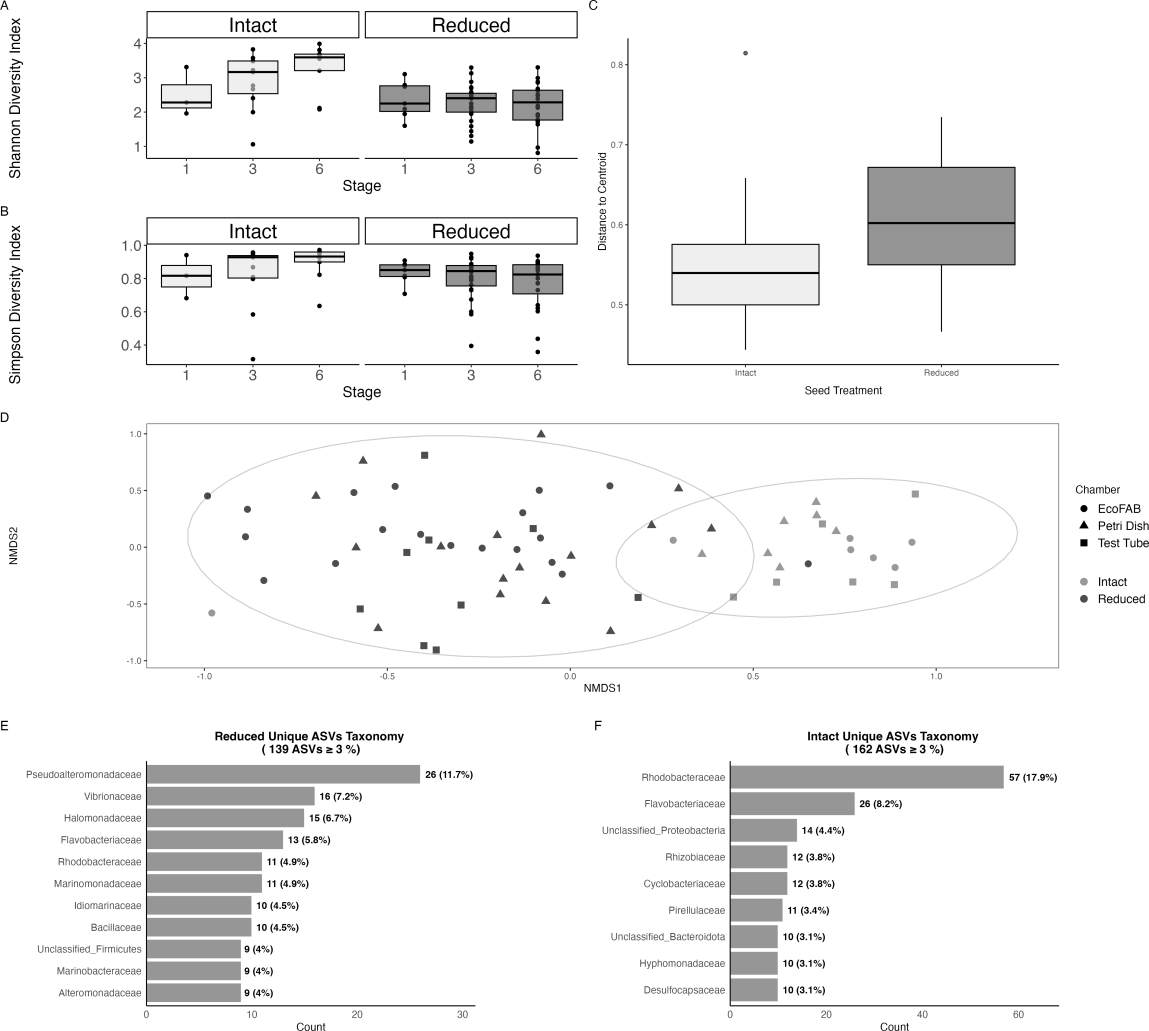
Eelgrass from surface-sterilized seeds (Reduced) has a lower alpha diversity and a higher beta-dispersion in their assembly than those from seeds with native microbiota (Intact). (**A**) Boxplots of Simpson and (**B**) Shannon alpha diversity of eelgrass microbiota across plant stages 1, 3, and 6 within seed treatment (reduced and intact). (**C**) Dispersion between plants with an intact versus reduced microbiota. (**D**) NMDS plot using Bray-Curtis dissimilarity of Hellinger-transformed ASV count data. Chamber type is designated by shape and color by seed treatment. (**E and F**) Horizontal bar charts displaying the family-level taxonomic composition of ASVs unique to each seed treatment group across developmental stages. Numbers in parentheses indicate the percentage of unique ASVs within each family. Families only representing more than 3% of the unique ASVs are shown.

To determine whether seed microbiota reduction altered microbiota assembly trajectories throughout plant development, we analyzed overall microbial richness and evenness (alpha diversity) as well as the differences in community composition among samples (beta diversity; Table 1). To provide a comprehensive analysis, we also included chamber effects in our model of microbiota composition, but focused our discussion on the effects of microbiota reduction. Since chamber type affects plant morphology (Fig. 2), its effect on microbiota composition is likely confounded by morphology-mediated changes. Chamber type and developmental stages of eelgrass did not impact the overall alpha diversity (Scheirer–Ray–Hare test; *P* > 0.05, FDR corrected), but seeds with reduced microbiota had significantly lower alpha diversity (Scheirer–Ray–Hare test: Simpson diversity *P* = 0.004, Shannon diversity *P* = 0.0001, FDR corrected; Fig. 4A and B). Microbiota reduction status explained 9.3% of the variation in community composition (Fig. 4C and D; Table 1; PERMANOVA, *P* < 0.05). All predictors, except the interaction effect between seed treatment and seedling stage, were also statistically significant. Additionally, seedlings from seeds with reduced microbiota showed greater microbiota dispersion across individuals than those from seeds with intact microbiota (Fig. 4C; PERMDISP, *P* = 0.0039).

**TABLE 1:** Seed treatment, chamber type, and plant developmental stage significantly affect the microbiota composition^*a*^

	df	Sum of squares	*R* ^2^	*F*	Pr (>*F*)^*b*^
Seed treatment	1	2.6784	0.09312	8.0058	**9.999e−05**
Chamber type	3	2.0401	0.07093	2.0326	**0.000200**
Stage	1	0.5383	0.01871	1.6088	**0.034997**
Seed treatment:chamber type	3	1.5070	0.05239	1.5015	**0.006599**
Seed treatment:stage	1	0.2919	0.01015	0.8725	0.633437
Chamber type:stage	2	0.9654	0.03356	1.4428	**0.031597**
Residual	62	20.7428	0.72114		
Total	73	28.7639	1		

^*a*^ Permutational multivariate analysis of variance (PERMANOVA) results based on Bray-Curtis dissimilarity of Hellinger-transformed ASV count data, testing the effects of seed treatment, chamber type, and plant developmental stage on microbiota community composition. The model used was ~ Seed treatment* Chamber + Seed treatment* Chamber + Stage + Chamber * Stage, including main effects and significant interactions. Seed treatment, chamber type, and development stage were significant factors, along with the Seed treatment × Chamber type and Chamber type × Stage interactions. Results are based on 10,000 permutations.

^*b*^ Bold values signify statistical significance (*P* < 0.05).

The observed reductions in alpha diversity and increases in inter-sample variation following microbiota reduction are comparable to what has been previously found with terrestrial plant studies (41, 69). The endophytic community retained within surface-sterilized seeds can move bidirectionally between internal and external plant compartments during seedling development and, in the absence of seed-derived epiphytes, face reduced competition for external colonization niches. In previous studies, seed surface sterilization of terrestrial plants has allowed for the successful establishment of targeted microbial additions (70, 71), presumably by reducing competition with the incumbent endophytic community. Similarly, in our system, the identity of potential surviving taxa after seed surface sterilization appeared to be stochastic, suggesting that establishment can be strongly influenced by introducing target taxa at high density in future inoculation experiments.

To identify taxa driving diversity differences between reduced and intact microbiota treatments, we first examined ASVs unique to each treatment (223 ASVs in reduced; 319 ASVs in intact). For families representing >3% of these unique ASVs, only Rhodobacteraceae and Flavobacteriaceae were observed in both treatments (Fig. 4D and E). Plants with intact microbiota had higher proportions of Rhodobacteraceae (17.9%) and Flavobacteriaceae (8.2%) among their unique ASVs, while these families comprised only 5.8 and 4.9% of unique ASVs in reduced microbiota, respectively. Both families are not only known to be associated with older eelgrass leaves, but are also found on many marine surfaces, which would explain their greater representation in intact seed microbiota (72). In addition, eelgrass that had intact microbiota had treatment-specific ASVs belonging to families typically associated with the rhizosphere and roots (i.e., Rhizobiaceae, Cyclobacteriaceae, and Desulfocapsaceae) or leaves (i.e., Hyphomonadaceae and Pirellulaceae) of the mother plant (73, 74). This microbial composition on the intact seed reflects the mother plant’s microbiota that the seeds would have been exposed to during maturation and initial release in the mesh bags and that was retained on the seed coat surface (75–78).

**Fig 5:**
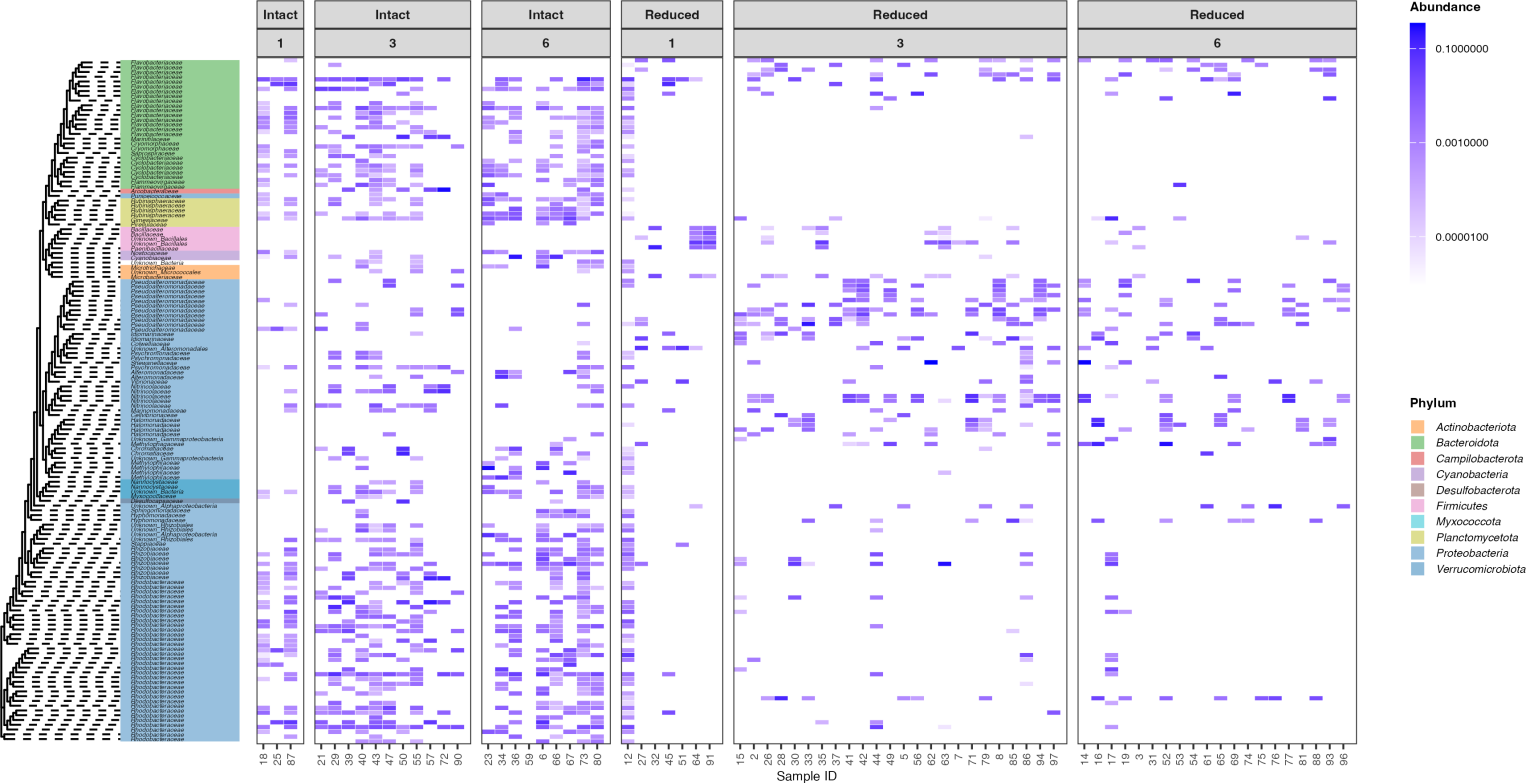
Eelgrass from native microbiota seeds (Intact) maintains higher relative abundances of taxa from Stage 1 onward unlike those from surface-sterilized seeds (Reduced). Heat map of the relative abundance (log10 transformed) of the 143 significantly different ASVs between reduced and intact plants at Stage 6, regardless of chamber type. Relative abundances are plotted across all developmental stages. Columns are grouped by whether the plant is from intact or reduced seeds and either Stage 3 or 6.

To further explore the trajectory of microbiota assembly between groups with reduced or intact microbiota as they near establishment, we analyzed plants at Stage 6 when both leaves and roots were fully developed. We used DESeq2 to identify individual ASVs that were significantly different in relative abundance between plants with reduced microbiota versus those with intact microbiota (log2 fold change >2; *P*-adjusted <0.05). Out of 2,358 ASVs, 38 had significantly higher relative abundances for eelgrass in the reduced microbiota treatment versus the intact group, while 105 ASVs had higher relative abundances for plants in the intact microbiota treatment (Fig. 5; Table S3).

ASVs enriched in eelgrass grown from seeds with reduced microbiota included members from the family Halomonadaceae (log2 fold change > 24), which includes many taxa with plant growth-promoting properties (79). For example, members of the Halomonadaceae contribute to plant salt tolerance, as these bacteria reduce the negative impacts of saline stress through the production of exopolysaccharides and other compounds, such as ectoine, that enhance terrestrial plant seed germination under high saline conditions (80–86). Due to being enriched in eelgrass from seeds with reduced microbiota and taxa reported as endophytes in other plants (87–89), we suggest that Halomonadaceae are important endophytic members of *Z. marina* during its early development.

Among the 105 ASVs enriched in the intact microbiota treatment, Rhizobiaceae emerged as a particularly notable family, with members showing substantial enrichment (log2 fold change 8.9–45) relative to the reduced group. The presence of Rhizobiaceae, along with the previously identified Rhodobacteraceae and Flavobacteriaceae, reflects the likely seed epiphytic microbial community derived from the mother plant material. Rhizobiaceae, which include Rhizobia, are known for nitrogen fixation and facilitate ammonium mobilization in the *Z. marina* rhizosphere (90). In general, there are higher amounts of nitrogen-fixing bacteria found in seagrass meadows compared to unvegetated sediment (91), with juvenile plants having higher relative abundances compared to adults (16). Therefore, bacteria with these capabilities may be important for seedling establishment. How seed epiphytes explicitly establish on the plant post-germination is largely unknown (but see War et al. for a full review), but their high relative abundances highlight their potential importance (92).

We observed a pattern throughout the plant development for the 143 ASVs flagged by our DESeq2 analysis: The microbiota on plants developing from seeds with intact microbiota established rapidly and remained stable throughout the experiment, at least with respect to the dominant taxa by Stage 6. However, when microbiota were reduced on seed surfaces, the resulting microbiota changed across plant development stages (Fig. 5). To further explore the stability of the microbiota between plant stages, we calculated Spearman’s rank correlation between stages 1 and 6 for the average abundance of each ASV for plants with reduced microbiota versus intact microbiota to see if ASVs that had high abundances at Stage 1 remain high at Stage 6. For intact microbiota, Spearman’s rho was 0.4391 (*P* = 4.12 e-08), suggesting that there was a moderate to strong ASV abundance consistency. Plants with reduced microbiota, however, had a Spearman’s rho of 0.09 (*P* = 0.264), suggesting no correlation between stages. Overall, our results support that we can alter the *Z. marina* microbiota with seed surface sterilization by decreasing the microbial diversity of the seed and altering the assembly dynamics of the microbiota throughout seed development.

### Eelgrass development causes shifts in the microbiota composition

**Fig 6:**
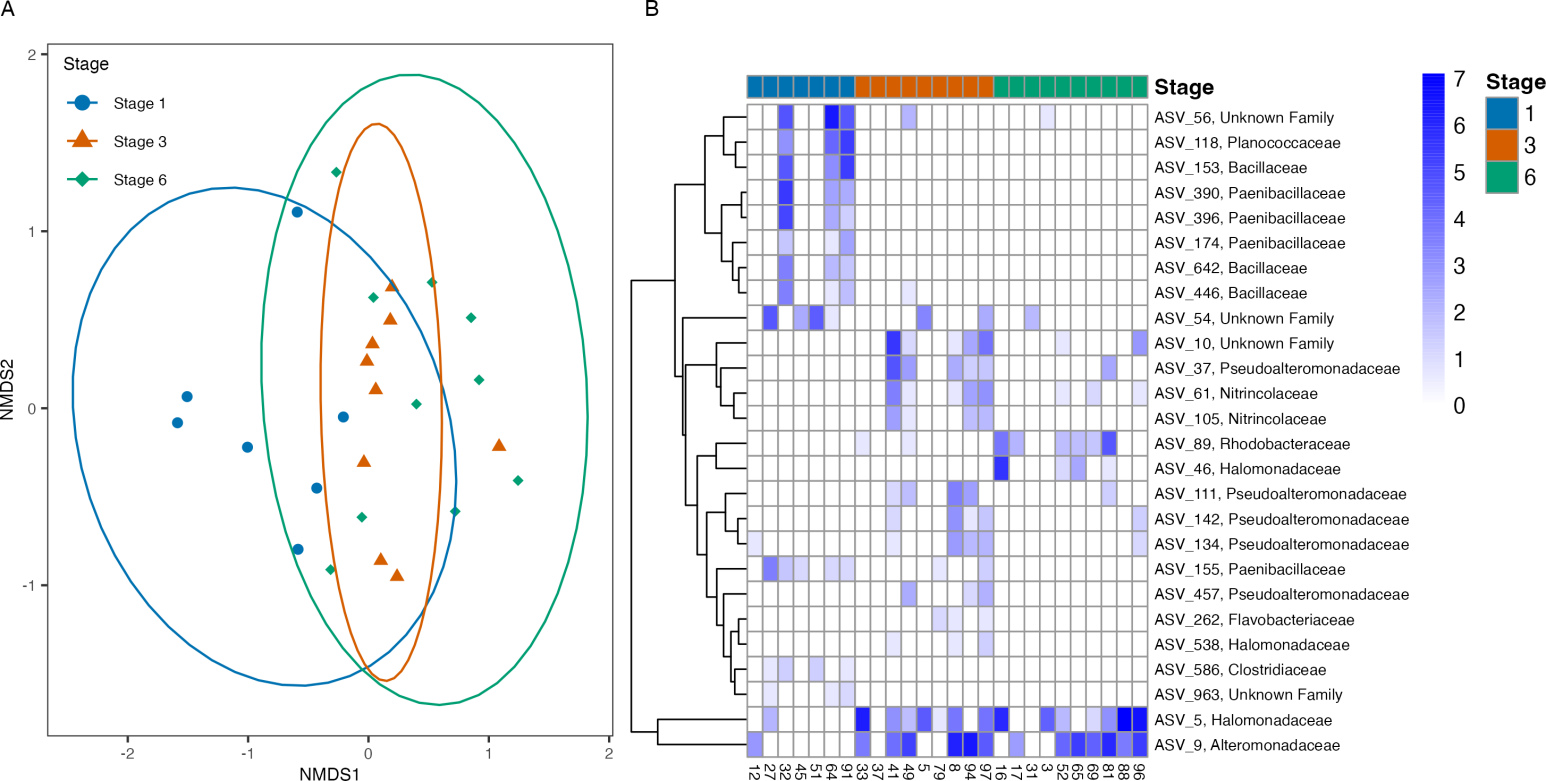
Microbiota composition shifts with host development. (**A**) NMDS plot based on Bray-Curtis dissimilarity of Hellinger-transformed ASV count data showing separation of microbiota across plant stages grown from seeds with reduced microbiota. (**B**) Heat map showing the relative abundance [log(1 + *x*) transformed] of the indicator species determined for each stage of the plant.

To investigate the trajectory of the microbiota during development, we restricted the analysis to reduced microbiota seeds grown in EcoFABs due to the interaction effect of stage and chamber type (*P* = 0.0146; Table 2; Stage 1 *n* = 7; Stage 3 *n* = 9; Stage 6 *n* = 10). By focusing on plants from seeds with reduced microbiota, we can determine how seed-derived endophytes assemble with limited impact of epiphytes derived from the environment. In addition, delving into this portion of the microbiome gives insight into potential bacterial members that are vertically transmitted from the mother to seeds. 16S rRNA gene amplicon sequencing of plants from surface-sterilized seeds revealed that microbiota composition significantly differed between stages 1, 3, and 6 (PERMANOVA *P* = 0.0043; Fig. 6A). We identified 26 ASVs in our indicator species analysis that are significantly associated with the three *Z. marina* developmental stages (Fig. 6B; Table S4). Of note, members of the Halomonadaceae family were found in stages 3 and 6. Halomonadaceae are expected to be increasing in abundance with plant development, likely providing support for osmotic regulation, stress signaling, and hormonal balance as seen for other plant hosts (93). Additionally, members of the Rhodobacteraceae family were found in Stage 6. Rhodobacteraceae are commonly associated as marine surface colonizers (24), but some species have been identified as endophytes for salt-tolerant plants *Salicornia europaea* and *Populus euphratica* Oliv., suggesting these bacteria may play specialized roles in salt tolerance during eelgrass seedling development. Salinity is a known key stressor influencing seedling establishment and growth in *Z. marina* during this period (29).

**TABLE 2:** Permutational multivariate analysis of variance (PERMANOVA) results based on Bray-Curtis dissimilarity of Hellinger-transformed ASV count data, testing the effects of chamber and stage on plants grown from seeds with reduced microbiota^*a*^

	df	Sum of squares	*R* ^2^	*F*	Pr (>*F*)^*b*^
Chamber type	3	1.9184	0.10083	1.8042	**0.0007**
Stage	1	0.3984	0.02094	1.1241	0.2817
Chamber type:stage	2	1.1137	0.05854	1.5711	**0.0146**
Residual	44	15.5950	0.81969		
Total	50	19.0255	1		

^*a*^ The model used was ~ Chamber Type * Stage. Results are based on 10,000 permutations.

^*b*^ Bold values signify statistical significance (*P* < 0.05).

Of the 12 indicator ASVs for Stage 1, 10 belonged to Bacillaceae and Paenibacillaceae. These same two families are dominant families (39 and 35.6%, respectively) in tomato seeds, and some species exhibit strong antifungal properties, suggesting their importance in seed protection (94). Furthermore, both families have shown to have capabilities to inhibit oomycetes (95, 96), which are common *Zostera* seed pathogens (35, 97). This stage-specific recruitment of functionally diverse bacterial families, including potentially protective and stress-tolerant taxa, indicates how marine plants may assemble specialized microbial partnerships to address the multifaceted challenges of early development. Seed-derived endophytes can drive a large portion of microbiota assembly in terrestrial plants, which is speculated to result from priority effects and vertical transmission (10, 14, 98–100); here, we found that similar processes can occur in seagrass seeds. This effect was particularly pronounced for seeds that had their initial microbiota reduced. Even if these form only a small component of the overall community in untreated seeds in nature, the putative key role these taxa play in stage-specific challenges may still affect the overall health of early life stages.

### Conclusion

In this study, we successfully manipulated the microbiota of eelgrass seeds in ways that influenced the microbiota composition throughout seedling development, but still allowed the development of plants through various seedling stages. Thus, we have established a standardized methodology for microbiota reduction in seagrass seedlings without altering host development. By developing an understanding of community composition patterns across plant stages and treatments, our work lays the foundation for future microcosm studies focusing on introducing beneficial microbes as synthetic communities to *Z. marina* seedlings. Future studies that expand our understanding of the functional capabilities of these organisms (via a metagenomics approach) or that include the characterization of the associated eukaryotic microbes would further progress our understanding of how microbiota assembly dynamics interact with the host.

Our findings also advance our understanding of the functional role of seed and seedling microbiota in eelgrass. We found that the reduction of the seed epiphytic microbial community does not have adverse effects on eelgrass early development. This could be because when the host is not under stress, the microbiota composition does not largely affect host function, as seen with the rhizosphere community of *Z. muelleri* (19). Indeed, a wide range of studies show increased mutualistic or facilitative effects as environmental stressors increase (e.g., references 101, 102). Stressors, such as high temperature, pathogens, or toxic sulfides in sediments, could all be ameliorated by eelgrass bacterial symbionts, but the effectiveness of this both in natural and restoration settings requires explicit testing.

Identifying both putative endophytes and epiphytes at different stages of plant growth is essential for experimentally testing the role of beneficial bacteria in plants (103–108). In our work, we show that eelgrass development had a stronger influence on endophytic than epiphytic community composition and that the epiphytic community had many overlapping taxa in microbiome studies of adult seagrass, which suggests vertical transmission (109). We further highlight families with potentially important roles: endophytic Halomondaceae for salt tolerance, epiphytic Rhizobiaceae for N acquisition, and endophytic Paenibacillaceae for pathogen protection. Further exploration of microbiome dynamics among taxa such as these is needed to determine which interactions have synergistic or antagonistic effects on the host (110).

Beyond identifying and testing bacterial candidates for desired host functions, we can leverage the EcoFAB 2.0 to explore plant metabolisms by manipulating abiotic and biotic conditions across plant compartments. While this study focuses on the bacterial members of the microbiome of the entire plant (leaf and root tissue together), EcoFAB 2.0’s dual-compartment setup also enables separate quantification of seagrass root and shoot microbiota shifts. This compartmentalized approach provides opportunities to examine plant–microbe interactions in both the phyllosphere and rhizosphere, ultimately leading to a more comprehensive understanding of how specific microbial members contribute to overall seagrass establishment, growth, and persistence.

## Data Availability

Read data sets are publicly available in NCBI under in BioProject no. PRJNA1332831. Input files and R code used for all analyses can be found at https://doi.org/10.5281/zenodo.20836820.

## SUPPLEMENTAL MATERIAL

Table S1 msystems.00722-26-s0001.csv Seagrass sample sizes across the entire experiment.

Table S2 msystems.00722-26-s0002.csv Seagrass seed observation data for all individual seeds.

Table S3 msystems.00722-26-s0003.csv DESeq2 analysis results.

Table S4 msystems.00722-26-s0004.csv Indicator species results.

ASM does not own the copyrights to Supplemental Material that may be linked to, or accessed through, an article. The authors have granted ASM a non-exclusive, world-wide license to publish the Supplemental Material files. Please contact the corresponding author directly for reuse.
